# Assessment of Thyroid Stiffness and Viscosity in Autoimmune Thyroiditis Using Novel Ultrasound-Based Techniques

**DOI:** 10.3390/biomedicines11030938

**Published:** 2023-03-17

**Authors:** Dana Stoian, Andreea Borlea, Ioan Sporea, Alexandru Popa, Luciana Moisa-Luca, Alina Popescu

**Affiliations:** 1 Division of Endocrinology, Department of Internal Medicine II, “Victor Babeș” University of Medicine and Pharmacy, E. Murgu Square, Nr. 2, 300041 Timisoara, Romania; 2 DrD Ultrasound Center, M. Cristea Nr. 9, 300029 Timisoara, Romania; 3 Centre for Molecular Research in Nephrology and Vascular Disease, “Victor Babeș” University of Medicine and Pharmacy, 300041 Timisoara, Romania; 4 Division of Gastroenterology and Hepatology, Department of Internal Medicine II, Victor Babeș University of Medicine and Pharmacy, 300041 Timisoara, Romania; 5 Advanced Regional Research Center in Gastroenterology and Hepatology, “Victor Babeș” University of Medicine and Pharmacy, 300041 Timisoara, Romania

**Keywords:** hashimoto, Vi PLUS, shear-wave, elastography, dispersion

## Abstract

The estimation of viscosity by measuring the shear-wave dispersion (SWD) using ultrasound 2D shear-wave elastography (SWE) is becoming more and more popular. Recent research suggests that SWD can be used in addition to 2D-SWE (shear-wave speed) to diagnose diffuse liver disease. Viscosity was studied for the assessment of normal thyroid tissue. This study aims to evaluate the use of viscosity measurements in patients with chronic autoimmune thyroiditis using the SuperSonic MACH^®^30 ultrasound machine (Hologic SuperSonic Imagine, Aix-en-Provence, France) which provides the Vi PLUS mode for viscosity and the 2D SWE PLUS mode for shear-wave speed measurements. Valid measurements were obtained in 308 cases, 153 with chronic autoimmune thyroiditis (CAT) and 155 with no thyroid pathology (95.95% feasibility of the methods). The differences between the healthy group and the CAT group were statistically significant both for Vi PLUS (2.5 ± 0.4 vs. 2.8 ± 0.5, *p* < 0.0001) and for 2D-SWE PLUS (13.5 ± 3.3 vs. 23.1 ± 8.3, *p* < 0.0001). The diagnostic performance was poor for Vi PLUS alone (AUC = 0.69; cut-off > 2.5 Pa·s, se = 68.6%; sp = 64.52%) and good for 2D-SWE PLUS alone (AUC = 0.861; cut-off > 18.4 kPa, se = 69.9%; sp = 92.2%). Vi PLUS correlated with 2D-SWE PLUS, with the presence of CAT, the thyroid volume, levothyroxine replacement therapy and age. Statistically significant differences were found between the CAT subgroup receiving thyroid replacement therapy and the subgroup without therapy: 24.74 ± 8.33 vs. 21.93 ± 8.12 kPa for 2D-SWE (*p* = 0.0380) and 3 ± 0.5 vs. 2.7 ± 0.4 Pa·s for Vi PLUS (*p* = 0.0193). Elastography-based methods improve the classic ultrasound evaluation: 2D-SWE PLUS performed somewhat better in distinguishing CAT from normal thyroid tissue, while Vi PLUS made a slightly better assessment regarding the functional status.

## 1. Introduction

Chronic autoimmune thyroiditis (CAT), also known as Hashimoto’s thyroiditis (HT), is an autoimmune condition of the thyroid gland, frequently characterized by a diffusely enlarged thyroid, lymphocytic infiltrate, fibrosis, and strongly positive serum levels of autoimmune antibodies: anti-thyroid peroxidase (ATPO) and/or anti-thyroglobulin (ATG) antibodies (Ab) [1,2]. In iodine-replete regions, CAT is the most frequent cause of hypothyroidism, but it can also be accompanied by euthyroid status, making the diagnosis less obvious [2,3]. The loss of immune tolerance, which leads to an autoimmune attack on thyroid tissue and the development of the disease, is thought to be caused by a confluence of hereditary predisposition and environmental factors. The disease’s origin may involve the protein CTL-4 or a cytotoxic T-lymphocyte antigen [4]. An early diagnosis is desired to minimize the effects of the disease and detect other possible autoimmune conditions, which may be present in about 20% of the patients with CAT in the context of a worldwide increase in autoimmune diseases [3,5]. Individuals who are experiencing CAT may have trouble concentrating and remembering things [6]. A contentious and poorly understood condition is Hashimoto encephalopathy, which is rare but severe. More than 80% of patients’ early symptoms have been described as cognitive impairment, and more than 90% of patients’ initial symptoms have been behavioral and personality abnormalities [7].

The ultrasound (US) evaluation may suggest the diagnosis and is helpful, particularly in subclinical forms of the disease [8]. In addition to the volume enlargement, the typical inhomogeneous, hypoechoic, pseudonodular ultrasound appearance of the disease is caused by immune cell-mediated cytotoxicity leading to thyrocyte destruction and infiltration of the parenchyma with inflammatory cells and eventually to fibrosis [9]. The Doppler findings are also useful—most cases present increased vascularity, which may represent a diagnostic clue [10]. Thyroid elastography has great potential in the US detection of autoimmune thyroiditis, given its pathological features and the presence of fibrosis. Radioiodine scans are not recommended for diagnostic purposes in CAT [8].

The SuperSonic MACH30 machine by Hologic provides a 2D imaging mode called viscosity Plane-wave UltraSound (Vi PLUS), a novel tool that allows the visualization and measurement of tissue viscosity inside an area of interest selected by the operator and quantified in Pascal seconds (Pa·s) [11,12]. A range of values for normal thyroid tissue was previously established [13,14], but the technique was not yet used in patients with thyroid diseases. The aim of this study is to assess the diagnostic value of Vi PLUS in detecting autoimmune thyroiditis and its predictive value for hypothyroidism in patients with CAT.

## 2. Material and Methods

### 2.1. Patients

A monocentric prospective study was performed for five months (May–September 2022), in which 321 patients were evaluated: 161 patients with known autoimmune thyroid disease who presented for periodical evaluation and 160 patients with no thyroid disease that presented for thyroid screening. People in the first group had autoimmune thyroiditis documented and confirmed by the presence of increased serum antithyroid antibodies (ATG and/or ATPO Abs). All patients in this group had TSH values in the normal range, either with or without levothyroxine supplementation. The patients in the second group had no family history of thyroid disease, normal TSH values and normal thyroid volume and US appearance on examination. Patients in both groups underwent a multiparameter US evaluation that included conventional, bidimensional shear-wave elastography (2D-SWE PLUS) and Vi PLUS measurements.

Patients with thyroid nodules detected on the US examination with abnormal thyroid stimulating hormone (TSH) levels and with a personal history of thyroid surgery or neck irradiation were excluded from the study, and advanced US-based evaluations were not performed in these cases. All of the patients lived in Timis County, Romania, which is historically regarded as an area rich in iodine [15].

The local ethics committee of the County Emergency Hospital “Pius Brinzeu” Timisoara (no. 235/2021) approved the study, which was conducted per the Declaration of Helsinki, updated in 2000, Edinburgh. All subjects gave their written informed consent before being enrolled in the study.

### 2.2. Ultrasound, 2D Shear-Wave and Vi PLUS Evaluation

All US-based methods were performed consecutively in the same evaluation, by the same examiner, with more than 20 years of experience in thyroid US and more than 4 years of experience in SWE techniques (D.S). For the examination, the patient was asked to lay in the supine position, with the neck in hyperextension facilitated by placing a pillow under the neck and coupling gel was used in a generous amount between the US probe and the skin to avoid pre-compression. The patient was instructed to breathe superficially in order to avoid movement artifacts.

The examination was carried out using the highly advanced SuperSonic MACH^®^30 ultrasound machine (Supersonic Imagine, Aix-en-Provence, France) and the UltraFast^TM^ image acquisition software. The initial evaluation in B-mode was performed with a high-frequency linear probe L18-5 (bandwidth: 5–18 MHz; footprint: 51 mm) in order to assess the thyroid volume and general appearance of the thyroid.

Immediately after the conventional US evaluation, the viscosity data were acquired simultaneously with stiffness measurements using the C6-1X single-crystal curved transducer (bandwidth: 1–6 MHz; footprint: 64 mm). The 2D-SWE PLUS and Vi PLUS modes were concomitantly activated, and stable images were acquired after 5 to 10 s of holding the probe still in order to avoid any artifacts.

**Figure 1:**
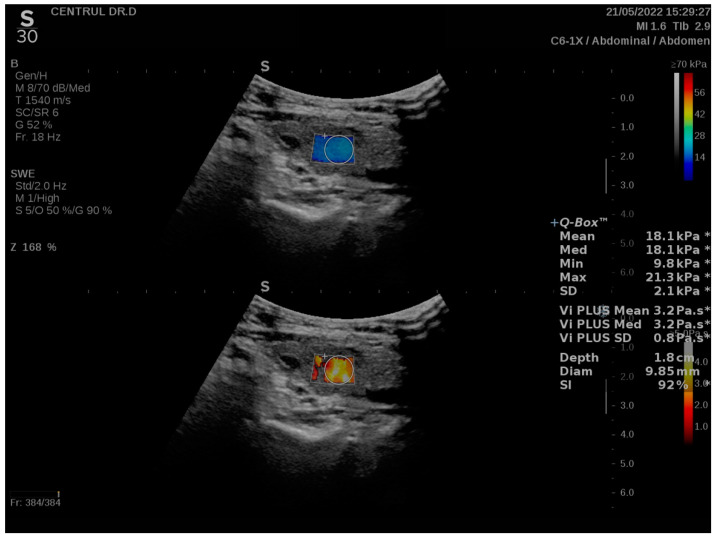
Shear-wave color map (**above**) and viscosity map (**below**) overlayed on the greyscale ultrasound image in a case of chronic autoimmune thyroiditis; shear-wave values (mean, median, minimum maximum and standard deviation estimated in kPa), viscosity values (mean, median and standard deviation estimated in Pa·s) and characteristics of the region of interest (depth in cm, diameter in mm and stability index in percentage) are displayed on the right side of the image; the SWE-based parameters are marked with “*”.

In addition to displaying the velocity fluctuations of the shear waves across different frequencies as a color map (white-yellow-red), the machine also offers a numerical value in pascal-seconds (Pa·s). The US machine is programmed to provide values between 1.0 and 5.0 Pa·s. With 2D-SWE PLUS, ultrafast imaging techniques are used to obtain a color map representing tissue stiffness alongside the Vi PLUS image; it ranges from dark blue-to-dark red according to the values of Young’s modulus measured in kPa. Additionally, a numerical value for tissue elasticity is given (kPa). A stability index (SI) greater than 90% was aimed at acquiring reliable images, as illustrated in Figure 1. 

The median value for 2D SWE PLUS and Vi PLUS from five consecutive valid measurements was considered in the final analysis.

### 2.3. Statistical Analysis

MedCalc V19.4 (MedCalc Software Ltd., Flanders, Belgium) was employed for the statistical analysis. For the demographic, anthropometric and clinical data, as well as for US findings, descriptive statistics were used. The Kolmogorov–Smirnov test was used to determine the distribution of the numerical variables. Mean and standard deviation were used to portray numerical variables with normal distributions, while median and range intervals were used to display variables with non-normal distributions. Figures and percentages were used to illustrate qualitative variables. In order to evaluate correlations for data with a normal distribution, Pearson’s correlation coefficient was calculated, and Spearman rank correlation (rho) was used to evaluate correlations for data with a non-normal distribution. In order to acquire the most suitable prediction model in both univariate and multivariate statistical studies, the predictors from the regression equations have been accepted in accordance with a repeating, stepwise backward algorithm. 95% confidence intervals (CI) were determined for each prediction test; a *p*-value of 0.05 or less was regarded as statistically significant.

## 3. Results

### 3.1. Baseline Characteristics and Feasibility of the SWE-Based Methods

Out of the 321 patients initially evaluated, 308 had valid measurements and were included in the final analysis. The mean age in the entire group was 39.5 ± 13.6, 80.2% being females. The subgroup distribution according to the presence of CAT is presented in Table 1.

**Figure 2:**
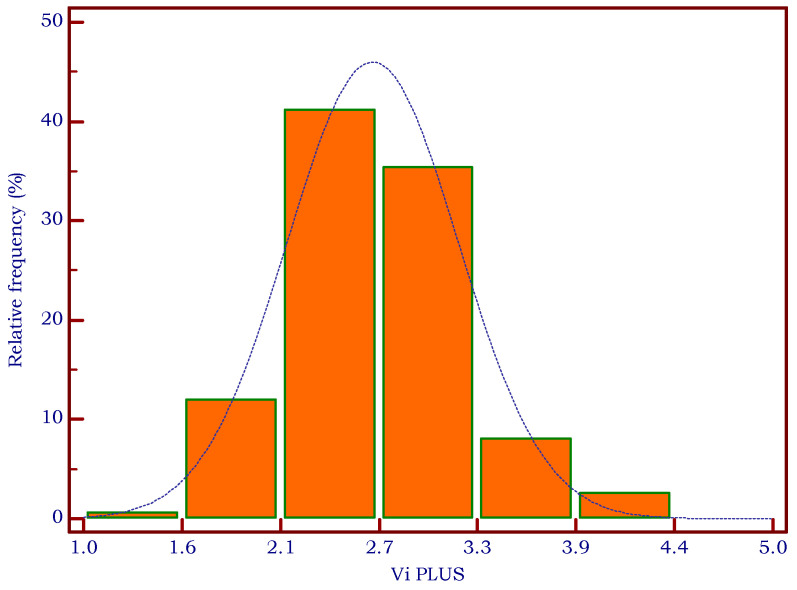
Relative frequency of mean Vi PLUS measurements.

**Figure 3:**
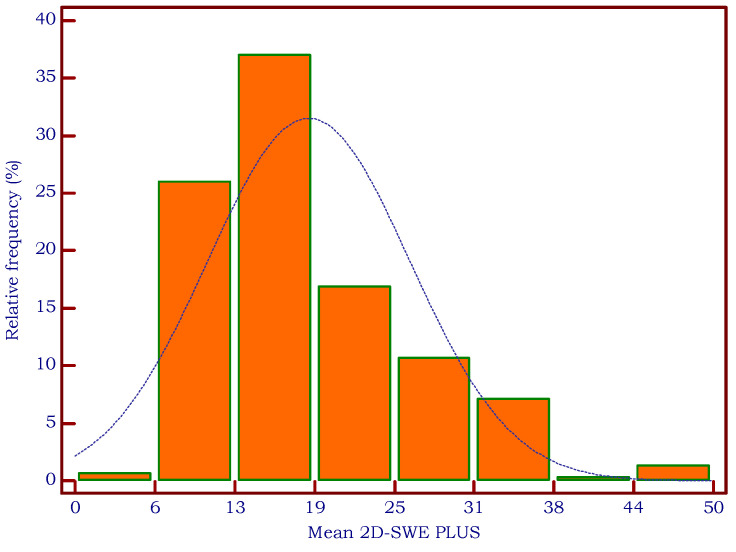
Relative frequency of mean 2D-SWE PLUS measurements.

In 13/321 patients, an incomplete filling of the color map or a SI of 90% prevented the acquisition of valid measurements with 2D-SWE PLUS and/or Vi PLUS, obtaining a very good feasibility of the methods of 95.95%. No differences were found for BMI (23.97 ± 3.8 vs. 25.87 ± 3.9, *p* = 0.0787) between patients with reliable and unreliable measurements. The histograms in Figure 2 and Figure 3 display the relative frequency of the elastography-based measures.

### 3.2. 2D SWE PLUS and Vi PLUS Values in Patients with Normal Thyroid and in Patients with Autoimmune Thyroiditis

The values obtained for 2D-SWE based measurements are displayed in Table 2.

**Figure 4:**
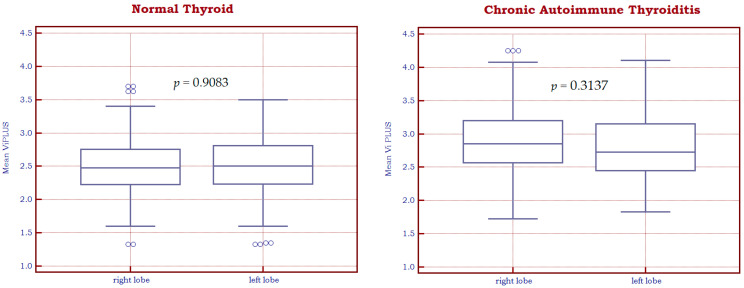
Box-and-whisker distribution plots representing Vi PLUS values of the left and right thyroid lobes for normal thyroid tissue (**left**) and autoimmune thyroiditis (**right**).

As displayed in Figure 4, no significant differences were found between the two thyroid lobes in terms of the Vi PLUS assessment, neither for healthy thyroid tissue (2.51 ± 0.46 Pa·s for the left lobe and 2.50 ± 0.45 Pa·s for the right one, *p* = 0.9083) nor for patients with CAT (2.81 ± 0.27 Pa·s for the left lobe and 2.87 ± 0.27 Pa·s for the right one, *p* = 0.3137).

**Figure 5:**
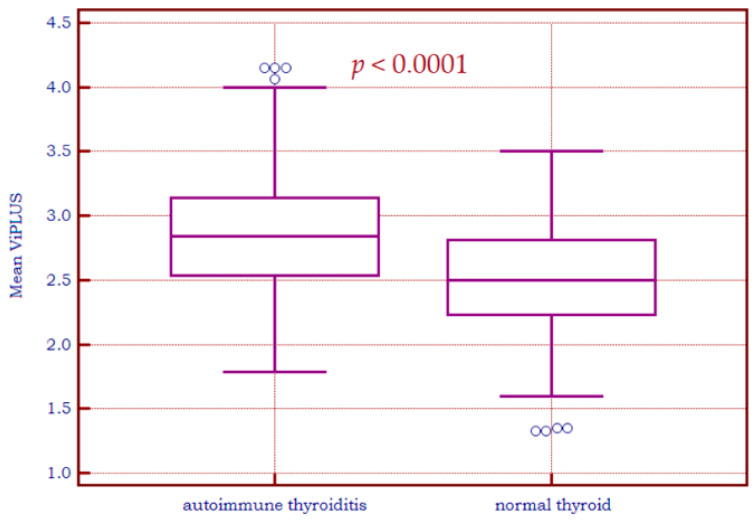
Box-and-whisker distribution plots representing Vi PLUS values in patients with autoimmune thyroiditis versus patients with a healthy thyroid.

The difference between means for Vi PLUS values in the healthy group and in the CAT group was statistically significant (2.8 ± 0.5 vs. 2.5 ± 0.4, *p* < 0.0001)—see Figure 5. However, there is a noticeable overlap between the two groups.

**Figure 6:**
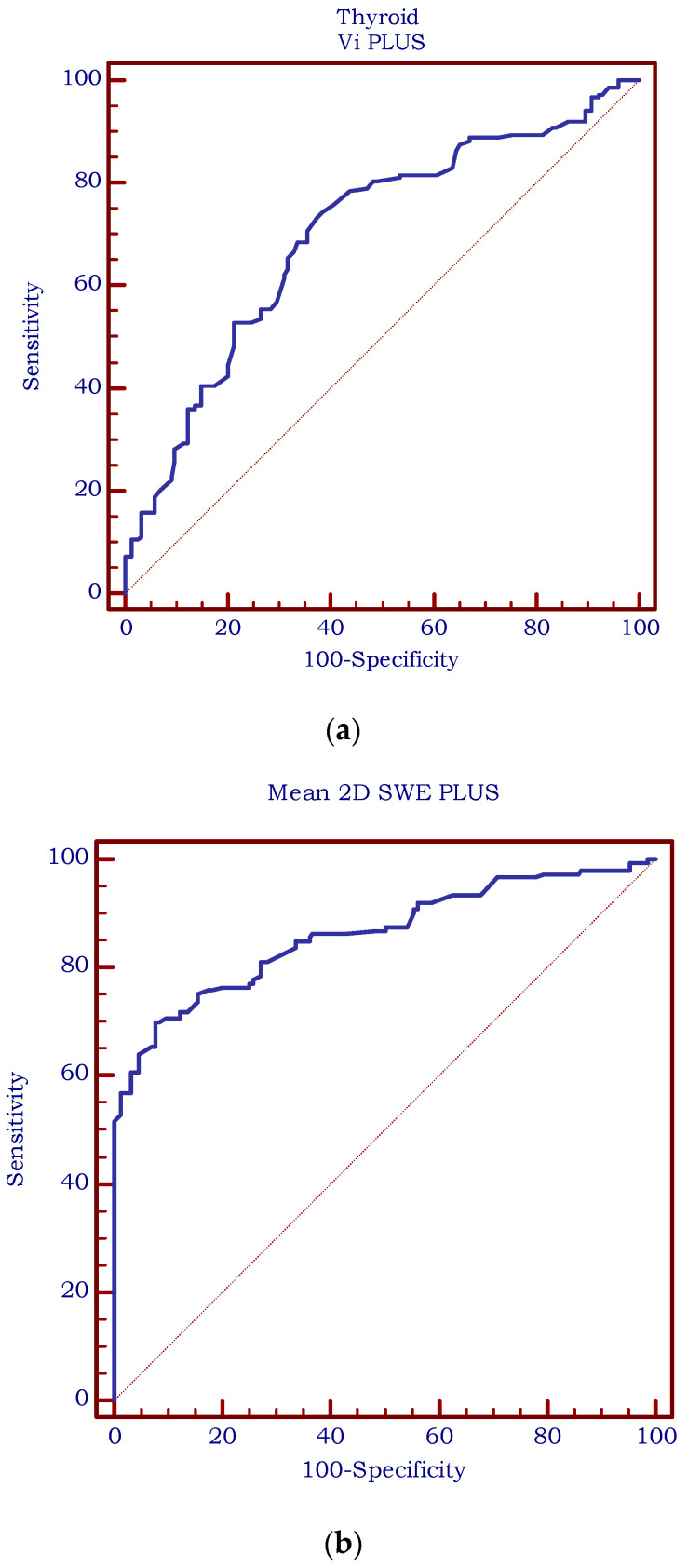
(**a**). Diagnostic performance of Vi PLUS for predicting chronic autoimmune thyroiditis (AUC = 0.69; cut-off value >2.5 Pa·s, sensitivity = 68.6%; specificity = 64.52%). (**b**). Diagnostic performance of 2D SWE PLUS for predicting chronic autoimmune thyroiditis (AUC = 0.861; cut-off value > 18.4 kPa, sensitivity = 69.9%; specificity = 92.2%).

The diagnostic performance was assessed using receiver operating characteristic (ROC) statistics. As for the area under the ROC Curve (AUC), a value of 0.69 was obtained for Vi PLUS with an optimal cut-off of >2.5 Pa·s (Se = 68.6%, Sp = 64.52%), as illustrated in Figure 6a. Figure 6b displays an AUC of 0.861 for 2D SWE PLUS (optimal cut-off value > 18.4 kPa, sensitivity = 69.9%; specificity = 92.2%).

### 3.3. 2D SWE PLUS and Vi PLUS Values in Patients with CAT with and without Levothyroxine Replacement

Differences between the CAT subgroup receiving thyroid replacement therapy and the subgroup without replacement therapy are detailed in Table 3. A statistically significant difference is observed between the mean elasticity and viscosity values of the two subgroups, but no differences were detected in terms of total thyroid volume.

**Figure 7:**
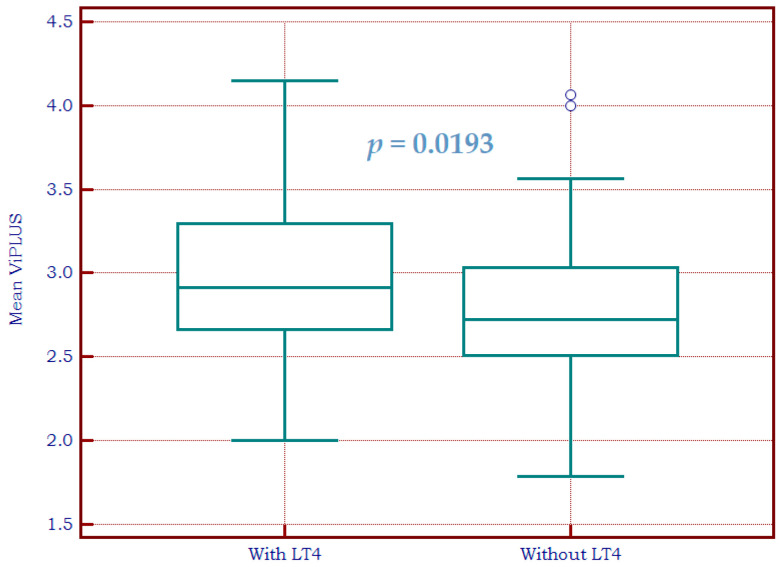
Box-and-whisker distribution plots representing Vi PLUS values in patients with autoimmune thyroiditis with and without levothyroxine treatment.

In the CAT group, the difference between the subgroup receiving levothyroxine treatment and the group without a need for replacement therapy is plotted in Figure 7.

**Figure 8:**
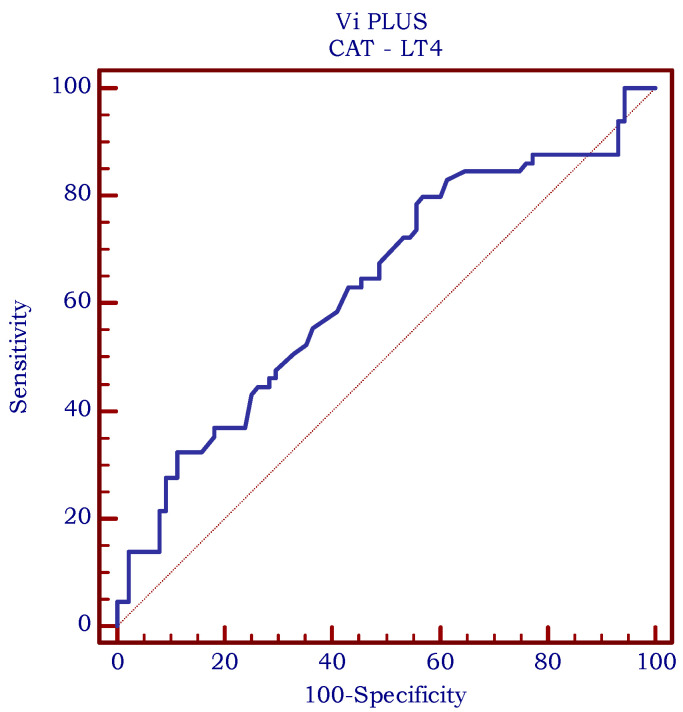
Diagnostic performance of Vi PLUS for predicting the need for levothyroxine supplementation in patients with chronic autoimmune thyroiditis (AUC = 0.63; cut-off value >2.6 Pa·s, sensitivity = 80%; specificity = 43.18%).

**Figure 9:**
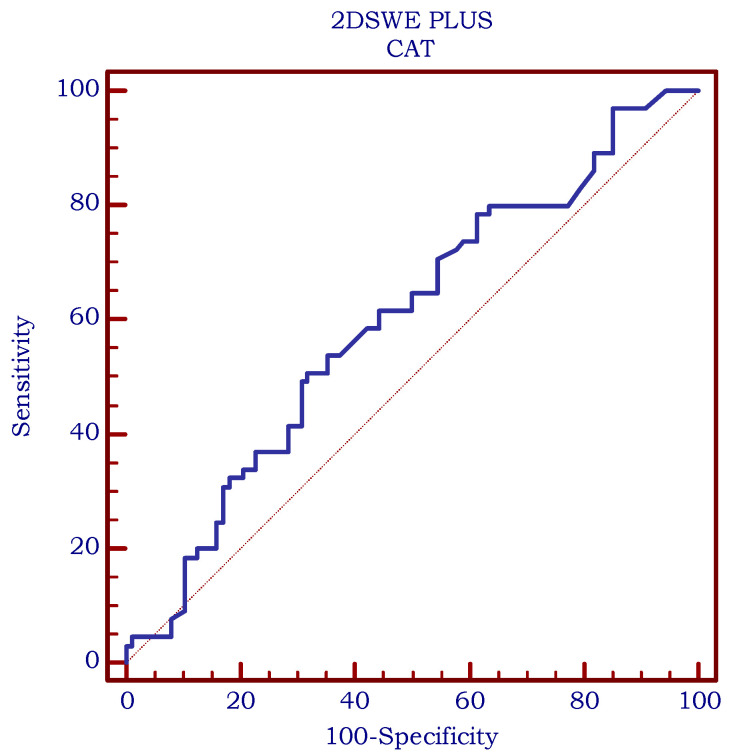
Diagnostic performance of 2D-SWE PLUS for predicting the need for levothyroxine supplementation in patients with chronic autoimmune thyroiditis (AUC = 0.59; cut-off value >24.5 kPa, sensitivity = 50.77%; specificity = 68.18%).

The AUC for detecting patients with LT4 needs in the CAT group is illustrated in Figure 8 for Vi PLUS and Figure 9 for 2D SWE PLUS. An AUC of 0.63 (0.55–0.70 CI) was obtained for the Vi PLUS measurement, and an optimal cut-off of >2.6 Pa·s (Se = 80%, Sp = 43.18%, NPV = 74.5%, PPV = 51%). For 2D SWE, an AUC of 0.59 (0.51–0.67 CI) was obtained and an optimal cut-off value of >24.5 kPa (Se = 50.77%, Sp = 68.18%, NPV = 62.5%, PPV = 54.1%).

### 3.4. Factors Affecting the Vi PLUS and 2D-SWE PLUS Assessment

In univariate regression, Vi PLUS was influenced by 2D SWE PLUS (*p* < 0.001), thyroid volume (*p* < 0.001) and the presence of CAT (*p* < 0.001). It was not influenced by the depth of measurements (*p* = 0.005), BMI (*p* = 0.702), LT4 treatment (*p* = 0.007), age (*p* = 0.005). In multivariate regression, SWE measurements were influenced by Vi PLUS (*p* = 0.0015), age (*p* = 0.0075), thyroid volume (*p* = 0.0013) and the presence of CAT (*p* < 0.001).

In univariate regression, 2D-SWE PLUS was also influenced by Vi PLUS (*p* < 0.001), thyroid volume (*p* < 0.001) and the presence of CAT (*p* < 0.001). It was not influenced by the depth of measurements (*p* = 0.084), BMI (*p* = 0.597), LT4 treatment (*p* = 0.038), age (*p* = 0.005). In multivariate regression, Vi PLUS was influenced only by SWE (*p* = 0.0015) and the presence of CAT (*p* = 0.007)

For Vi PLUS, a moderate positive correlation was obtained with 2D-SWE PLUS (r = 0.510, *p* < 0.0001), a weak positive correlation with the presence of CAT (r = 0.340, *p* < 0.0001), the thyroid volume (r = 0.210, *p* = 0.0002) and LT4 replacement (r = 0.218, *p* = 0.0068). A very weak positive correlation was obtained between Vi PLUS and age (r = 0.160, *p* = 0.0050).

Additionally, 2D-SWE PLUS correlated strongly with the presence of CAT (r = 0.607, *p* < 0.0001), moderately with the thyroid volume (r = 0.408, *p* < 0.0001), weakly with gender (r = −0.203, *p* = 0.0003), age (r = 0.338, *p* < 0.0001) and very weakly with LT4 replacement (r = 0.168, *p* = 0.0381). Correlations between the analyzed parameters are detailed in Table 4.

When considering only the group with CAT, in univariate regression, both SWE and ViPLUS values were influenced by LT4 treatment (*p* = 0.038 and *p* = 0.007).

## 4. Discussion

Elastography represents an excellent tool, especially in the detection of thyroid nodules that are suspicious of malignancy [16,17,18,19], but also in assessing the presence of autoimmune thyroiditis [20] or Graves’ disease [21]. Assessing mechanical tissue properties is an estimation rather than an exact measurement. In 2D SWE, the thyroid stiffness measured using the shear-wave speed is regarded as a non-invasive marker of fibrosis, while the thyroid viscosity measured using the dispersion slope is expected to become a marker of inflammation [22]. These modern imaging methods, developed to date by Canon and Supersonic Imagine, take into account the algorithmic properties of tissue viscosity.

CAT defines a chronic thyroid inflammation with an etiopathogenesis that is still incompletely defined, although it was first described more than a century ago. The advancement of CAT and the degree of thyroid fibrosis are both strongly correlated with the rise in thyroid stiffness [23]. Although the cytomorphological characteristics of thyroiditis are highly overlapping, fine-needle aspiration and cytological investigations do not represent the exclusive method of diagnosis [24].

Normal values for thyroid elasticity were found to be around 9.0  ±  11.3 kPa and 19.5  ±  7.6 kPa in the literature [25], and 13.5 ± 3.3 kPa in our study. There is currently enough evidence that thyroid tissue stiffness has increased values in patients with CAT compared to the normal thyroid tissue, with mean 2D-SWE values ranging from 19.5  ±  6.8 kPa to 36.15  ±  18.7 kPa [24,25,26], even for pediatric patients [27]. In our study, the mean value was comparable for SWE: 23.1 ± 8.3 kPa. We found that 2D SWE values correlate with the thyroid volume (r = 0.408, *p* < 0.0001), with gender (r = −0.203, *p* = 0.0003), age (r = 0.338, *p* < 0.0001) and with LT4 replacement (r = 0.168, *p* = 0.0381). With SWE techniques, Ruchala et al. found that CAT raises the stiffness of the thyroid tissue, which is unaffected by the later addition of LT4 therapy [24]. Margi et al. discovered a positive relationship between serum ATPO titer and tissue stiffness [28].

In a study by Rianna et al., experimental models were used to examine thyroid viscoelastic properties, and they found that independent of substrate stiffness, malignant thyroid cells had the tendency to have constant viscosity and elasticity properties. In contrast, normal thyroid cells have a similar trend of increasing rigidity with increasing substrate stiffness, as does dynamic viscosity [29].

As a first step in evaluating thyroid viscosity, the viscosity of normal thyroid tissue was previously evaluated recently by two studies. It was established to be around 2.42 ± 0.41 Pa·s in the first study, which evaluated 121 healthy subjects [13] and 2.63 ± 0.47 Pa·s in the second study on 85 healthy volunteers [14]. Both studies estimated thyroid viscosity using the Vi PLUS method provided by the SuperSonic device and a curvilinear probe. Other superficial organs were investigated with the Vi PLUS technique: the parotid gland (PG) and submandibular glands (SMG) had mean values of 2.13 ± 0.23 Pa·s and 2.44 ± 0.35 Pa·s, respectively [11]. It is interesting to note that the values for submandibular gland viscosity determined by the studies described above and ours are very close.

We obtained significant differences between means in the two groups: normal thyroid and CAT (*p* < 0.0001). Vi PLUS had a good diagnostic performance in detecting CAT, as shown by the value of the AUC and an optimal cut-off was established to be above 2.5 Pa·s (Se = 68.6%, Sp = 64.52%). There are no other published data to compare, as viscosity was not yet evaluated in any thyroid pathologies.

So far, viscosity has been studied only in some applications. In peripheral muscle, both 2D-SWE and viscosity parameters presented statistically significant differences between pre-and post-contraction settings [30]. Together with shear-wave elastography, tissue viscosity was also found to be useful in monitoring renal allograft in transplanted patients and detecting chronic injury [31]. In patients with chronic liver disease, spleen stiffness had better diagnostic quality than spleen viscosity, although they both correlated with the presence of liver disease [32]. Other authors also used non-traditional imaging techniques in Hashimoto’s disease that confirm the correlation with thyroid hormone levels. In a recent study, magnetic resonance spectroscopy was used to detect metabolic alterations in CAT patients’ otherwise normal-appearing brains [6]

We identified some limitations of our study. The Vi PLUS module could only be used with a curvilinear transducer at the time of the study enrollment, which was a significant constraint on our investigation. We expect even better results in the future, with the implementation of this technique on linear probes that are able to characterize surface structures more precisely. Nevertheless, we did obtain valid measurements, with a SI of over 90%. Additionally, in our study, the levels of ATPO and ATG Abs were not correlated with the US, SWE and Vi PLUS findings, given that the laboratory assays were performed in different settings and thus were considered not to be uniform.

As previously suggested for liver disease, in clinical practice, we expect to be able to use viscosity assessment to better distinguish between inflammatory processes, necrosis, desmoplastic transformation and fibrosis, which all show increased stiffness on elastography assessment. In the future, there is potential for combining these novel techniques in detecting different types of diffuse thyroid disease, such as Graves’ disease, chronic autoimmune thyroiditis, subacute thyroiditis, and other forms of destructive thyroiditis. Viscosity measurements could also possibly estimate the functional status of the thyroid, as suggested by the differences found in this study between patients with and without LT4 supplementation.

## 5. Conclusions

Our research has demonstrated that elastography-based methods add diagnostic power to classic ultrasound evaluation. They provide a rapid, easy-to-learn, non-invasive and accessible means of detecting thyroid autoimmunity in a clinical setting. 2D-SWE PLUS performed somewhat better in distinguishing CAT from normal thyroid tissue, while Vi PLUS made a slightly better assessment regarding the functional status.

## Figures and Tables

**Table 1:** Baseline characteristics for the patients with and without CAT.

	Normal Thyroid	CAT	Significance Level (*p*)
Number of evaluated patients	160	161	-
Valid Vi PLUS…measurements	155 (96.8%)	153 (95%)	0.5950
Female gender	104 (67%)	143 (93.4%)	<0.0001
Age (years)	34.6 ± 12	44.4 ± 13.3	<0.0001
Thyroid volume (ml)	12.74 ± 3.27	17.37 ± 6.16	<0.0001
Number of patients with LT4—RT	0 (0%)	65 (40.3%)	-
BMI (kg/m^2^)	23.8 ± 3.8	24 ± 3.7	0.6402

CAT—chronic autoimmune thyroiditis; Vi PLUS—viscosity plane-wave ultrasound; LT4—RT = levothyroxine replacement therapy; BMI—body mass index.

**Table 2:** Viscosity and shear-wave measurements of patients with and without CAT.

US-Based Parameter		Normal Thyroid	CAT	Significance Level (*p*)
Mean 2D-SWE PLUS (kPa)	Mean ± SD	13.5 ± 3.3	23.1 ± 8.3	<0.0001
	Median (95%CI)	13.8 (12.28–14.10)	22.9 (20.00–23.96)	
	Min	6	8	
	Max	22.1	46.4	
ViPLUS (Pa·s)	Mean ± SD	2.5 ± 0.4	2.8 ± 0.5	<0.0001
	Median (95%CI)	2.5	2.8	
	Min	1.33	1.7	
	Max	3.58	4.1	
Depth (cm)	Mean ± SD	1.6 ± 0.3	1.7 ± 0.3	0.0037
	Median (95%CI)	1.6	1.7	
	Min	1.1	1.2	
	Max	2.6	2.6	

CAT—chronic autoimmune thyroiditis; 2D-SWE PLUS—two-dimensional shear-wave elastography plane-wave ultrasound; Vi PLUS—viscosity plane-wave ultrasound; SD—standard deviation; CI—confidence interval.

**Table 3:** Differences of means for the 2D-SWE-based parameters (2D-SWE PLUS and Vi PLUS) for the group with and without levothyroxine replacement therapy.

	CAT with LT4 Replacement	CAT without LT4 Replacement	Significance Level (*p)*
Mean 2D-SWE PLUS (kPa)	24.74 ± 8.33	21.93 ± 8.12	0.0380
ViPLUS (Pa·s)	3 ± 0.5	2.7 ± 0.4	0.0193
Thyroid volume	18 ± 6.7	16.8 ± 5.7	0.2470

LT4—levothyroxine; CAT—chronic autoimmune thyroiditis; 2D-SWE PLUS—two-dimensional shear-wave elastography plane-wave ultrasound; Vi PLUS—viscosity plane-wave ultrasound.

**Table 4:** Correlation table using Pearson correlation coefficient.

		Mean Vi Plus	Mean 2D SWE	Cat	Depth	BMI	LT4 Replacement	Gender	AGE
Mean 2D SWE	r	0.510							
	*p*	<0.0001							
	n	308							
Cat	r	0.340	0.607						
	*p*	<0.0001	<0.0001						
	n	308	308						
Depth	r	0.112	0.098	0.101					
	*p*	0.0499	0.0845	0.0774					
	n	308	308	308					
BMI	r	−0.020	0.030	0.024	0.102				
	*p*	0.7204	0.5966	0.6757	0.0750				
	n	308	308	308	308				
LT4 Replacement	r	0.218	0.168	0.000	−0.020	0.049			
	*p*	0.0068	0.0381	-	0.8071	0.5502			
	n	153	153	-	153	153			
Gender	r	−0.069	−0.203	−0.331	0.233	0.021	−0.013		
	*p*	0.2258	0.0003	<0.0001	<0.0001	0.7189	0.8705		
	n	308	308	308	308	308	153		
AGE	r	0.160	0.338	0.361	0.060	0.035	0.252	−0.226	
	*p*	0.0050	<0.0001	<0.0001	0.2945	0.5456	0.0017	0.0001	
	n	308	308	308	308	308	153	308	
Volume	r	0.210	0.408	0.426	0.089	0.047	0.094	−0.152	0.136
	*p*	0.0002	<0.0001	<0.0001	0.1183	0.4092	0.2470	0.0074	0.0172
	n	308	308	308	308	308	153	308	308

2D-SWE PLUS—two-dimensional shear-wave elastography plane-wave ultrasound; CAT—chronic autoimmune thyroiditis; BMI—body mass index; Vi PLUS—viscosity plane-wave ultrasound; LT4—levothyroxine; r—Pearson correlation coefficient; *p*—probability value; n—number of patients in the group.

## References

[1] Fink H., Hintze G. (2010). Die Autoimmunthyreoiditis (Hashimoto-Thyreoiditis): Aktuelle diagnostik und therapie [Autoimmune thyroiditis (Hashimoto’s thyroiditis): Current diagnostics and therapy]. Med. Klin..

[2] Hu X., Chen Y., Shen Y., Tian R., Sheng Y., Que H. (2022). Global prevalence and epidemiological trends of Hashimoto’s thyroiditis in adults: A systematic review and meta-analysis. Front. Public Health.

[3] Ragusa F., Fallahi P., Elia G., Gonnella D., Paparo S.R., Giusti C., Churilov L.P., Ferrari S.M., Antonelli A. (2019). Hashimotos’ thyroiditis: Epidemiology, pathogenesis, clinic and therapy. Best Pract. Res. Clin. Endocrinol. Metab..

[4] Feldkamp J. (2009). Autoimmunthyreoiditis: Diagnostik und Therapie. DMW—Dtsch. Med. Wochenschr..

[5] Lerner A., Jeremias P., Matthias T. (2015). The World Incidence and Prevalence of Autoimmune Diseases is Increasing. Int. J. Celiac Dis..

[6] Bladowska J., Waliszewska-Prosół M., Ejma M., Sąsiadek M. (2019). The metabolic alterations within the normal appearing brain in patients with Hashimoto’s thyroiditis are correlated with hormonal changes. Metab. Brain Dis..

[7] Waliszewska-Prosół M., Ejma M. (2022). Hashimoto Encephalopathy—Still More Questions than Answers. Cells.

[8] Dighe M., Barr R., Bojunga J., Cantisani V., Cristina Chammas M., Cosgrove D., Cui X.W., Dong Y., Fenner F., Radzina M. (2017). Thyroid Ultrasound: State of the Art Part 1—Thyroid Ultrasound reporting and Diffuse Thyroid Diseases. Med. Ultrason.

[9] Wu G., Zou D., Cai H., Liu Y. (2016). Ultrasonography in the diagnosis of Hashimoto’s thyroiditis. FBL.

[10] Mahmoud R., Azeem K.M., Sayed A.S.A., Ali F.M. (2022). Role of ultrasound and Doppler findings as a predictor of thyroid hormonal levels in cases of Hashimoto thyroiditis. Beni Suef Univ. J. Basic Appl. Sci..

[11] Muntean D., Lenghel M., Ciurea A., Dudea S. (2022). Viscosity Plane-wave UltraSound (ViPLUS) in the assessment of parotid and submandibular glands in healthy subjects—preliminary results. Med. Ultrason.

[12] Popa A., Bende F., Șirli R., Popescu A., Bâldea V., Lupușoru R., Cotrău R., Fofiu R., Foncea C., Sporea I. (2021). Quantification of Liver Fibrosis, Steatosis, and Viscosity Using Multiparametric Ultrasound in Patients with Non-Alcoholic Liver Disease: A “Real-Life” Cohort Study. Diagnostics.

[13] Stoian D., Moisa L., Taban L., Sporea I., Popa A., Bende F., Popescu A., Borlea A. (2022). Quantification of Thyroid Viscosity in Healthy Subjects Using Ultrasound Shear Wave Dispersion (Viscosity PLUS). Diagnostics.

[14] Petea-Balea D.R., Solomon C., Muntean D.D., Dulgheriu I.T., Silaghi C.A., Dudea S.M. (2022). Viscosity Plane-Wave UltraSound (Vi PLUS) in the Evaluation of Thyroid Gland in Healthy Volunteers&mdash;A Preliminary Study. Diagnostics.

[15] Simescu M., Popescu R., Ionitiu D., Zbranca E., Grecu E., Marinescu E., Tintea L., Nicolaescu E., Purice M., Popa M., Delange F., Dunn J.T., Glinoer D. (1993). The Status of Iodine Nutrition in Romania. odine Deficiency in Europe: A Continuing Concern.

[16] Cantisani V., D’Andrea V., Biancari F., Medvedyeva O., di Segni M., Olive M., Patrizi G., Redler A., De Antoni E.E., Masciangelo R. (2012). Prospective evaluation of multiparametric ultrasound and quantitative elastosonography in the differential diagnosis of benign and malignant thyroid nodules: Preliminary experience. Eur. J. Radiol..

[17] Cantisani V., Lodise P., di Rocco G., Grazhdani H., Giannotti D., Patrizi G., Patrizi G., Redler A., De Antoni E.E., Masciangelo R. (2015). Diagnostic Accuracy and Interobserver Agreement of Quasistatic Ultrasound Elastography in the Diagnosis of Thyroid Nodules TT—Diagnostische Genauigkeit und Interobserver-Übereinstimmung der Quasistatischen-Ultraschall-Elastografie bei der Diagnose von. Ultraschall Med..

[18] Swan K.Z., Nielsen V.E., Bonnema S.J. (2021). Evaluation of thyroid nodules by shear wave elastography: A review of current knowledge. J. Endocrinol. Investig..

[19] Borlea A., Borcan F., Sporea I., Dehelean C.A., Negrea R., Cotoi L., Stoian D. (2020). TI-RADS Diagnostic Performance: Which Algorithm is Superior and How Elastography and 4D Vascularity Improve the Malignancy Risk Assessment. Diagnostics.

[20] Cepeha C.M., Paul C., Borlea A., Borcan F., Fofiu R., Dehelean C.A., Stoian D. (2020). The Value of Strain Elastography in Predicting Autoimmune Thyroiditis. Diagnostics.

[21] Hefeda M.M. (2019). Value of the New Elastography Technique using Acoustic Radiation Force Impulse in Differentiation between Hashimoto’s Thyroiditis and Graves’ Disease. J. Clin. Imaging Sci..

[22] Sugimoto K., Moriyasu F., Oshiro H., Takeuchi H., Yoshimasu Y., Kasai Y., Itoi T. (2019). Clinical utilization of shear wave dispersion imaging in diffuse liver disease. Ultrasonography.

[23] Ruchała M., Szmyt K., Sławek S., Zybek A., Szczepanek-Parulska E. (2014). Ultrasound sonoelastography in the evaluation of thyroiditis and autoimmune thyroid disease. Endokrynol. Pol..

[24] Ruchala M., Szczepanek-Parulska E., Zybek A., Moczko J., Czarnywojtek A., Kaminski G., Sowinski J. (2012). The role of sonoelastography in acute, subacute and chronic thyroiditis: A novel application of the method. Eur. J. Endocrinol..

[25] Kara T., Ateş F., Durmaz M.S., Akyürek N., Durmaz F.G., Özbakır B., Öztürk M. (2020). Assessment of thyroid gland elasticity with shear-wave elastography in Hashimoto’s thyroiditis patients. J. Ultrasound.

[26] Liu J., Zhang Y., Ji Y., Wan Q., Dun G. (2018). The value of shear wave elastography in diffuse thyroid disease. Clin. Imaging.

[27] Kandemirli S.G., Bayramoglu Z., Caliskan E., Sari Z.N.A., Adaletli I. (2018). Quantitative assessment of thyroid gland elasticity with shear-wave elastography in pediatric patients with Hashimoto’s thyroiditis. J. Med. Ultrason.

[28] Magri F., Chytiris S., Capelli V., Alessi S., Nalon E., Rotondi M., Cassibba S., Calliada F., Chiovato L. (2012). Shear wave elastography in the diagnosis of thyroid nodules: Feasibility in the case of coexistent chronic autoimmune Hashimoto’s thyroiditis. Clin. Endocrinol..

[29] Rianna C., Radmacher M. (2017). Comparison of viscoelastic properties of cancer and normal thyroid cells on different stiffness substrates. Eur. Biophys. J..

[30] Dulgheriu I.T., Solomon C., Muntean D.D., Petea-Balea R., Lenghel M., Ciurea A.I., Dudea S.M. (2022). Shear-Wave Elastography and Viscosity PLUS for the Assessment of Peripheric Muscles in Healthy Subjects: A Pre- and Post-Contraction Study. Diagnostics.

[31] Maralescu F.M., Bende F., Sporea I., Popescu A., Șirli R., Schiller A., Petrica L., Moga T.V., Mare R., Grosu I. (2022). Assessment of Renal Allograft Stiffness and Viscosity Using 2D SWE PLUS and Vi PLUS Measures—A Pilot Study. J. Clin. Med..

[32] Hirooka M., Koizumi Y., Nakamura Y., Yano R., Okazaki Y., Sunago K., Imai Y., Watanabe T., Yoshida O., Tokumoto Y. (2023). Spleen stiffness in patients with chronic liver disease evaluated by 2-D shear wave elastography with ultrasound multiparametric imaging. Hepatol. Res..

